# Persistent Depressive Symptoms and the Changes in Serum Cystatin C Levels in the Elderly: A Longitudinal Cohort Study

**DOI:** 10.3389/fpsyt.2022.917082

**Published:** 2022-06-03

**Authors:** Tiandong Han, Li Zhang, Weixing Jiang, Lei Wang

**Affiliations:** ^1^Department of Urology, Beijing Friendship Hospital, Capital Medical University, Beijing, China; ^2^Department of Anesthesiology, Beijing Friendship Hospital, Capital Medical University, Beijing, China

**Keywords:** persistent depressive symptoms, serum cystatin C, longitudinal cohort study, the HRS, renal function

## Abstract

**Background:**

The burden of depression in the elderly is increasing worldwide with global aging. However, there is still a lack of research on the relationship between depressive symptoms and the progression of renal function. Our aim is to evaluate the longitudinal association between baseline depressive symptoms and the changes in serum cystatin C levels over 10 years' follow-up period.

**Methods:**

We used longitudinal data from the Health and Retirement Study (HRS), an existing community based nationally representative aging cohort study which enrolled individuals over age 50 in the USA. Depressive symptoms were determined using an eight-item version of the Center for Epidemiologic Studies Depression Scale (CESD) at wave 7 (2004) and wave 8 (2006). Persistent depressive symptoms were defined as both CESD scores measured at waves 7 and 8 were ≥3; episodic depressive symptoms were defined as CESD scores ≥3 at wave 7 or wave 8. A linear mixed model was used to evaluate the correlation between baseline depressive symptoms and future changes in cystatin C levels.

**Results:**

The mean age of the 7,642 participants was 63.8 ± 10.8 years, and 60.9% were women. Among the participants, 1,240 (16.2%) had episodic depressive symptoms and 778 (10.2%) had persistent depressive symptoms. Compared with participants with no depressive symptoms at both waves, a significant increase in serum cystatin C levels was found among those with persistent depressive symptoms.

**Conclusions:**

Our results showed that baseline persistent depressive symptoms were significantly associated with an increased rate of serum cystatin C levels. The level of serum cystatin C should be monitored in the elderly with persistent depressive symptoms.

## Introduction

In worldwide, all the governments need to face the social problem of depression in older people, as it might lead to increasing burdens and costs to individuals and society. To explore the modifiable risk factors and effective prevention for depression is needed. Accumulating evidences from studies suggest that depression can regulate the response of angiogenesis and immune state, which may cause increased mortality (1). Although depression is a common health problem in the middle-aged and elderly (2, 3), it is reported that depression is also common in patients with Chronic Kidney Disease (CKD) (4).

However, in recent years, there is limited research on the progress of renal function failure and depression in the elderly. Recently, a cross-sectional study reported the association between depression and renal function measured by estimated glomerular filtration rate (eGFR) (5). In addition, the manifestations of depression in various comorbidities are obesity, diabetes, and cardiovascular diseases. It may further predict poor renal prognosis (6, 7). However, the longitudinal relationship between baseline depression or depressive symptoms and future age-related renal impairment (from mild to moderate) is unclear.

Although serum or plasma creatinine have become the most commonly used serum marker of renal function, these are often misleading in the elderly, because muscle mass declines with aging, creatinine metabolism could be also changed (8). Cystatin C is a member of the type 2 cystatin superfamily of cysteine protease inhibitors. It is a small protein produced by almost all human cells and can be obtained in all body fluids (9). With freely filtered through the glomerulus, then reabsorbed and completely catabolized without secretion or subsequent reabsorption into the circulation (10), the characteristic is less dependent on muscle mass than creatinine, so that Cystatin C may be more accurate in kidney function's evaluation (9, 11).

The objectives of the current analysis were 1) to investigate whether depressive symptoms are associated with the levels of serum cystatin C at the baseline; and 2) to develop a better understanding of the potential relevance between baseline depressive symptoms and future changes of cystatin C levels in the elderly.

## Methods

### Study Population

We used longitudinal data from the Health and Retirement Study (HRS), an existing community based nationally representative aging cohort study which is freely available to all researchers. Since 1992, HRS has randomly recruited individuals over the age of 50 in the United States through a multistage regional probability sampling design (12). The cohort was in accordance with the Helsinki Declaration and approved by the Institutional Review Committee for Health Sciences and behavioral sciences at the University of Michigan (HUM00061128). Written informed consent was obtained from all participants.

**Figure 1:**
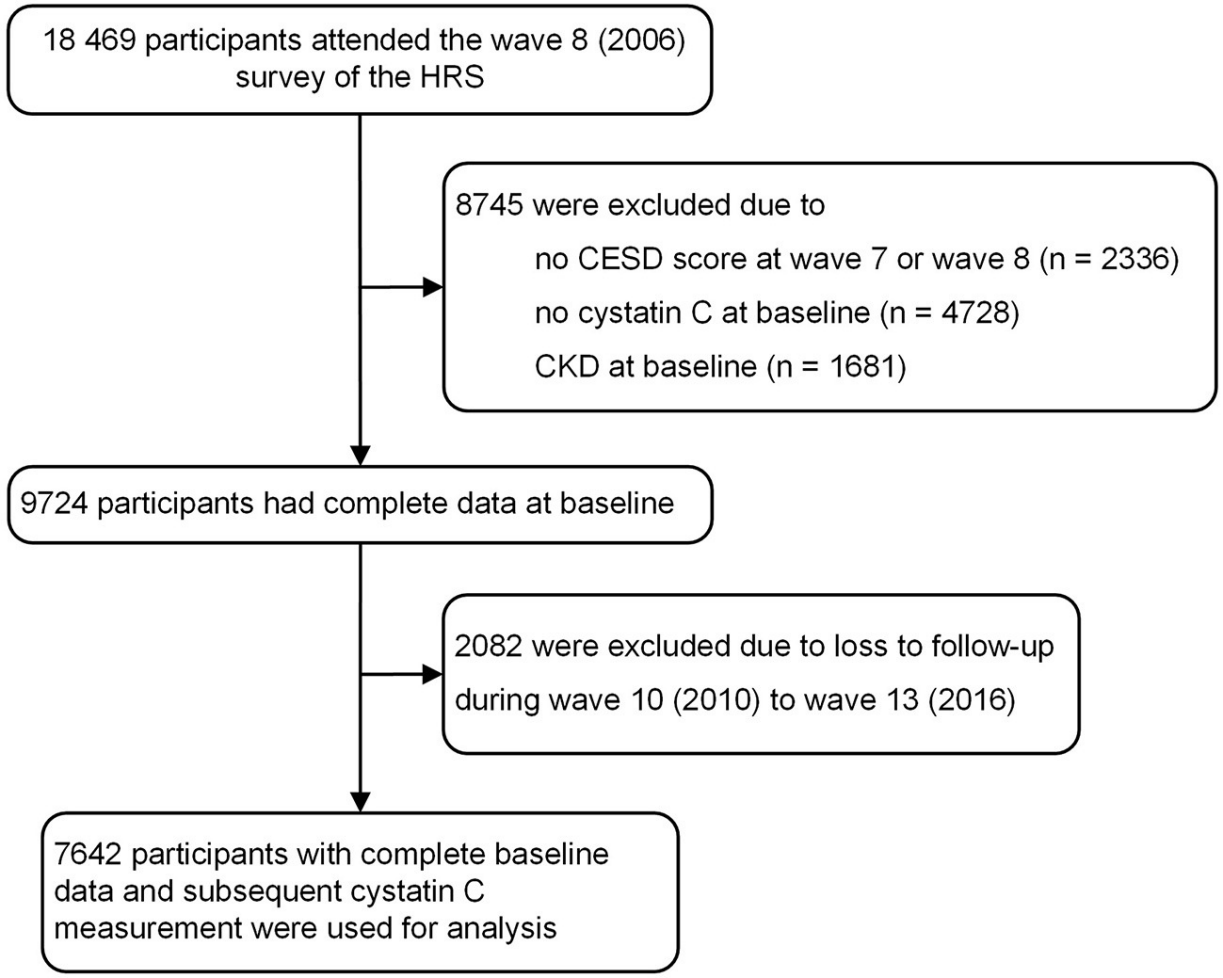
Flow chart of participant selection for this study.

Figure 1 shows the flow chart of the current participant analysis. In our study, wave 8 (2006) in HRS was regarded as the baseline. A total of 18,469 participants participated in the wave 8 survey of HRS. The exclusion standard included: (1) missing completion at the wave 7 or wave 8 of the Center for Epidemiologic Studies Depression Scale (CESD) scores, (2) no data of serum cystatin C at the baseline, (3) history of CKD at baseline, or (4) additional 2,082 individuals without follow-up during wave 10 to wave 13.

### Depressive Symptoms

Depressive symptoms were measured by the eight-item version of the Center for Epidemiological Studies Depression scale (CESD-8). This is a widely used self-report measure of depressive symptoms, which has been used in many population-based studies to identify people at risk of depression (13). Participants were asked to think about the past time and feelings, and then distinguish whether the following statements are more similar to them: you feel depressed; You find everything difficult; Your sleep is disturbed; You are happy; You feel lonely; You enjoy life; You feel sad; You can't go. To select Yes or No. The CESD version has internal consistency and factor structure, which is equivalent to the longer version of the scale (14). Although there were scores ≥4, according to the relevant survey, people were considered to have depressive symptoms (15, 16). In our study, depressive symptoms were defined as three or more of eight, which is a threshold used to indicate the clinical diagnosis of depression (17). People with depressive symptoms at wave 7 or wave 8 were classified as having episodic depressive symptoms, and those with depressive symptoms in both waves were classified as persistent depressive symptoms.

### Measurement of Serum Cystatin C

In the wave 8 (2006) survey of HRS, half of the participants were randomly preselected for blood collection, wave 9 (2008) included the other half. So that both wave surveys decided the baseline serum cystatin C data. Blood specimens were collected by dried blood spot. Serum cystatin C was detected in these two waves using a BNII nephelometer (Siemens, Inc., Deerfield, IL) at the University of Vermont. The equivalent value of serum cystatin C in the national health and Nutrition Examination Survey recommended by HRS researchers was used in our analysis.

### Definition of CKD and All-Cause Death

Renal function was defined by using the CKD Epidemiology Collaboration (CKD-EPI) equation, the new CKD-EPI equation was first published in 2012 with cystatin C alone or combined with creatinine (10). The most basic application of cystatin C is for GFR estimation. The 2012 Kidney Disease Improving Global Outcomes (KDIGO) recommended that cystatin C be measured in adults with creatinine eGFR from 45–60 mL/min/1.73m^2^ but lack of other markers of renal damage to confirm the diagnosis of CKD (18). The mortality data were taken from the tracker file with verification from family members.

### Assessment of Covariates

Covariates included clinical and demographic characteristics. Clinical characteristics included hypertension and diabetes. As for demographic characteristics, sex, race, age (years), and body mass index (kg/m^2^) were selected for our analysis.

Hypertension was identified as a patient's systolic blood pressure ≥ 140 mmHg and / or diastolic blood pressure ≥ 90 mmHg, or if the participant was currently using relative antihypertensive drugs. Body mass index was defined as weight (kg)/height^2^ (m^2^). The definition for Diabetes as hemoglobin A1c >6.5%, fasting blood glucose >7 mmol/L, or currently used for anti-diabetic treatment (16).

### Statistical Analysis

Characteristics of the overall sample were described using means and standard deviations (SD) or median (quartile range) for continuous data, and *n* (%) for categorical data.

Linear regression models were used to evaluate the cross-sectional associations between baseline status of depressive symptoms and serum cystatin C levels. Then, we used linear mixed model to perform longitudinal associations between sum of CES-D scores and the changes of serum cystatin C levels. We also ran longitudinal analyses to explore the association between baseline status of depressive symptoms and future changes of serum cystatin C levels, using participants without depressive symptoms as the reference. Linear mixed models use all available data during follow-up, considering that repeated measurements of the same participant are interrelated, and can deal with missing data (16). Cox regression models were used to analyze the longitudinal associations of baseline persistent depressive symptoms with incident CKD and all-cause mortality. Model 1 was adjusted for age and gender, and Model 2 was further adjusted for race, body mass index, hypertension, and diabetes.

All analyses were performed by using SAS (version 9.4; SAS Institute Inc). All tests were bilateral, the value is 0.05 as the threshold of statistical significance.

## Results

### Participant Selection and Characteristics

A total of 18,469 participants were included. Detailed process of participant selection was shown in Figure 1. Among these, 7,642 participants with complete baseline data and subsequent cystatin C measurement were used for our analysis. 778 participants (10.2%) were classified as experiencing persistent depressive symptoms, the other 1,240 participants (16.2%) were classified as experiencing episodic depressive symptoms.

Table 1 shows the baseline characteristics of participants according to the baseline status of depressive symptoms. The mean age of included participants for the HRS was 63.8 ± 10.8 years. In generally speaking, participants who reported a longer duration of depressive symptoms were significantly less well characterized than those who did not. Study participants reporting longer duration had higher body mass index, were more likely to be female, and had a higher prevalence of hypertension and diabetes mellitus.

**Table 1:** Characteristics of the study participants at baseline.

**Characteristic**	**Depressive symptoms at waves 7 and 8**			***P* for trend^***a***^**
	**Persistent (*n* = 778)**	**Episodic (*n* = 1,240)**	**No (*n* = 5,624)**	
Women (%)	556 (71.5)	864 (69.7)	3,231 (57.5)	<0.001
White (%)	573 (73.7)	961 (77.5)	4,833 (85.9)	<0.001
Age (Year)	63.0 ± 10.0	63.5 ± 9.4	65.3 ± 9.2	<0.001
Body mass index (kg/m2)	31.1 ± 6.8	29.8 ± 5.8	29.0 ± 5.4	<0.001
Hypertension (%)	440 (56.6)	626 (50.6)	2,592 (46.1)	<0.001
Diabetes (%)	186 (23.9)	241 (19.5)	737 (13.1)	<0.001
Cystatin C (mg/L)	0.97 (0.84–1.11)	0.94 (0.82–1.11)	0.93 (0.80–1.07)	0.006

*Data are presented as mean ± standard deviation, n (%), or median (quartiles 1–3).*

*^a^Calculated by using a linear regression analysis or χ^2^ test for trend*.

### Cross-Sectional Association Between Depressive Symptoms and Serum Cystatin C

**Figure 2:**
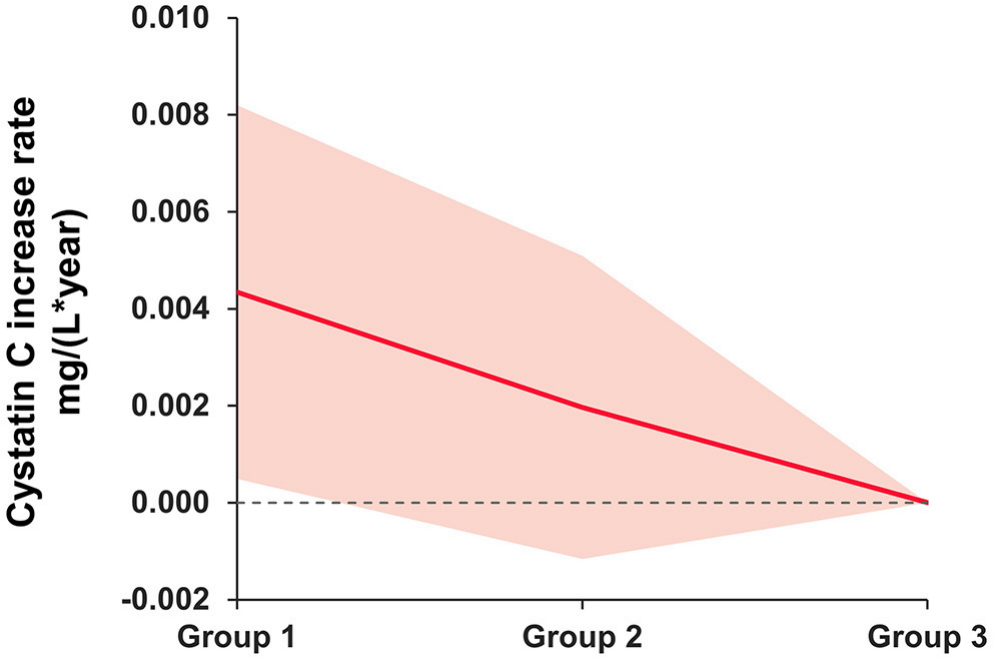
Mean difference rate of change in serum cystatin C (mg/L) between baseline depressive symptom groups. Group 1 as persistent depressive symptoms. Group 2 as episodic depressive symptoms. Group 3 as no depressive symptoms. The group 3 was the reference group. Solid lines represent mean differences in serum cystatin C (mg/L) after adjusting for age, sex, race, body mass index, hypertension, and diabetes. The shadows represent the 95% CIs. The detailed results are presented in Table 3.

As seen in Figure 2, solid lines represent mean differences in serum cystatin C (mg/L) after adjusting for age, sex, race, body mass index, hypertension, and diabetes. The shadows represent the 95% confidence intervals (CI). Compared with participants with no depressive symptoms at both waves, a significant increase in serum cystatin C levels was found among those with persistent depressive symptoms.

### Longitudinal Association Between Depressive Symptoms and Serum Cystatin C

As shown in Table 2, the longitudinal correlation between the sum of CESD scores and the rate of change in serum cystatin C. After multivariable adjustment, an increase of one unit in the total score of CESD was associated with a faster increase in serum cystatin C levels in patients with persistent and episodic depressive symptoms (0.004 mg/L, 95% CI: 0.001 to 0.008, *P* = 0.025; 0.002 mg/L, 95% CI: −0.001 to 0.005, *P* = 0.288), respectively, compared with participants without depressive symptoms. Besides, our analyses found that per 3-point increase in CESD scores was associated with a higher rate of change in serum cystatin C levels (0.003 mg/L, 95% CI: 0.001 to 0.005, *P* = 0.003), after multivariable adjustment.

**Table 2:** Association between baseline depressive symptoms and rate of change in serum cystatin C (mg/L): longitudinal analyses using linear mixed models.

**Depressive symptoms at waves 7 and 8**	**Model 1** ^**a**^		**Model 2** ^**b**^	
	**β (95% CI)**	***P-*value**	**β (95% CI)**	***P-*value**
Persistent	0.004 (0.001 to 0.008)	0.025	0.004 (0.000 to 0.008)	0.027
Episodic	0.002 (−0.001 to 0.005)	0.288	0.002 (−0.001 to 0.005)	0.218
No	Reference		Reference	
Per 3-point increase^c^	0.003 (0.001 to 0.005)	0.004	0.003 (0.001 to 0.005)	0.004

*^a^Model 1: adjusted for age and sex.*

*^b^Model 2: adjusted for age, sex, race, body mass index, hypertension, and diabetes.*

*^c^Per 3-point increasement of the mean CESD score at wave 7 and wave 8*.

### Longitudinal Associations of Depressive Symptoms With Incident CKD and All-Cause Death

As shown in Table 3, the risks of CKD and all-cause death in participants with persistent depressive were significantly or marginally significantly higher, compared with those without depressive symptoms. Similarly, CESD scores, as a continuous variable, was always significantly correlated with new onset CKD and all-cause death after multivariate adjustment.

**Table 3:** Association between baseline depressive symptoms and risk of incident CKD and all-cause mortality.

		**Model 1** ^**a**^		**Model 2** ^**b**^	
	**Events/Total**	**HR (95%CI)**	***P*-value**	**HR (95%CI)**	***P*-value**
**CKD**					
Persistent	243/492	1.512 (1.318 to 1.735)	<0.001	1.283 (1.061 to 1.552)	0.010
Episodic	339/1,240	1.268 (1.128 to 1.425)	<0.001	1.093 (0.928 to 1.288)	0.286
No	1,327/5,624	Reference		Reference	
Per 3-point increase^c^		1.350 (1.258 to 1.449)	<0.001	1.178 (1.065 to 1.304)	0.002
**All-cause death**					
Persistent	148/492	1.567 (1.314 to 1.868)	<0.001	1.267 (0.991 to 1.620)	0.059
Episodic	205/1,240	1.289 (1.106 to 1.503)	0.001	1.226 (0.997 to 1.507)	0.054
No	896/5,624	Reference		Reference	
Per 3-point increase^c^		1.365 (1.242 to 1.501)	<0.001	1.246 (1.093 to 1.420)	<0.001

*^a^Model 1: adjusted for age and sex.*

*^b^Model 2: adjusted for age, sex, race, body mass index, hypertension, and diabetes.*

*^c^Per 3-point increasement of the mean CESD score at wave 7 and wave 8*.

## Discussion

This ongoing cohort study of an elderly American populations investigate the temporal association between episodic or persistent depressive symptoms and the level of cystatin C in a large general population based on previous assessments of depressive symptoms. After extensive adjustment for covariates, persistent depressive symptoms were significantly associated with the change of kidney dysfunction, incident CKD, and all-cause mortality. These associations were not related to gender, age, race, body mass index, hypertension, and diabetes.

Emerging evidences suggest that depression and CKD may both affect most of the world's population, depression is more common in CKD patients than without CKD (19). In general, depressive symptoms can cause active inflammation and impaired immune response which may lead to poor clinical outcomes (20). Even if all patients with depression receive the best treatment of evidence-based intervention, residual symptoms and dysfunction are still common (21). Therefore, some previous studies have evaluated relationship between depressive symptoms and rapid eGFR decrease or progression to renal failure (22–26). Considering the adverse clinical results caused by depressive symptoms, the high prevalence of these symptoms in elderly maintained the importance of our study.

There are many mechanisms for the relationship between depressive symptoms and serum cystatin C. Although etiology and pathogenesis of depression are not fully understood, there are consistent and strong empirical evidences that neuronal injury and immune inflammation are important factors related to depression (27–31). Cystatin C is an important endogenous inhibitor of cysteine protease activity (32), which is also closely related to the nerve injury and immune inflammation (33, 34). Another possible mechanism is that cystatin C is close to oxidative stress, which can play a significant role in the process of depression (35). Last but not least, apoptosis may be an important risk factor for depression, it can also influence on the level of Cystatin C by changing the concentration of active caspinase-9 protein (36, 37). However, most investigations by now laid the emphasis on the relationship about higher cystatin C concentration appear to present with high risk of depression (38, 39). Different from previous studies, in this large cohort study of older Americans, persistent depressive symptoms were significantly associated with elevated serum cystatin C levels.

To our knowledge, this study is the largest general population-based investigation exploring the relationship between depressive symptoms and level of serum cystatin C with a long-term follow-up of 10 years. In our research, we showed that patients with higher depression events correlated with higher serum levels of cystatin C, then had an increased CES-D score at baseline. Our results imply that the prospective association between depressive symptoms and rapid kidney function decline in the general population, serum cystatin C might be a more sensitive biomarker for CKD.

What is more, we guaranteed comprehensive follow-up and strict adjudication of depression events, so that the results are reliable. Then, the determination method of serum cystatin C assay chosen for this study is both widely available and stable (coefficient of variation of precision <5%). The serum index is simple, reliable, and convenient for clinical promotion.

Several potential limitations need to be mentioned. The study sample comes from a single cohort, and the sample size may be larger. Additionally, the time courses of cystatin C levels were not evaluated and amended, especially the information for serum cystatin C level was absent in wave 7. By evaluating the time course of cystatin C, further research is necessary to consolidate the clinical significance of depressive symptoms. In addition, HRS enrolled only middle-aged and older persons, and as such, we do not know whether this association is suitable for younger person.

Several researchers have already evaluated the potential link between cystatin C levels and depression (38–40). A study which enrolled 1,440 Chinese elders (>60 years old) found a harmful relationship between high serum cystatin C levels and risk of depression (38). Another prospective cohort study of 11,847 Chinese people (>45 years old) demonstrated that high levels of serum cystatin C were associated with an increased risk of depression (40). They computed the risk of depression by using modified Poission regression models, found that the association between serum cystatin C levels and the risk of depression remained significant after adjusting for multiple covariance. Consistent with the above findings, our results show that serum cystatin C not only is associated with depression, but also severity of depressive symptoms is closely related to change of serum cystatin C. This association persists after controlling for multiple potentially confounding variables. We indicate that cystatin C should be monitored in the pathogenesis of depression among all the kidney function markers.

## Conclusions

Our results showed that baseline persistent depressive symptoms were significantly correlated with an increased rate of serum cystatin C levels. The level of serum cystatin C should be monitored in the elderly with persistent depressive symptoms. Potential mechanisms of the relationship between kidney dysfunction and depression need to be further characterized. If further confirmed, our data could be the evidence for depressive symptoms' screening and effective psychosocial intervention to improve the primary prevention of CKD and all-cause death.

## Data Availability Statement

The original contributions presented in the study are included in the article/supplementary material, further inquiries can be directed to the corresponding author.

## Ethics Statement

The studies involving human participants were reviewed and approved by the Institutional Review Committee for Health Sciences and Behavioral Sciences at the University of Michigan (HUM00061128). The patients/participants provided their written informed consent to participate in this study. Written informed consent was obtained from the individual(s) for the publication of any potentially identifiable images or data included in this article.

## Author Contributions

TH and LZ conceived the study and designed the statistical analyses. TH, LZ, and LW did the statistical analyses and prepared the draft of the manuscript. TH, WJ, LZ, and LW substantively revised the manuscript. All authors contributed to the article and approved the submitted version.

## Funding

The National Natural Science Foundation of China (81901125) have contributed to analysis and interpretation of data.

## Conflict of Interest

The authors declare that the research was conducted in the absence of any commercial or financial relationships that could be construed as a potential conflict of interest.

## Publisher's Note

All claims expressed in this article are solely those of the authors and do not necessarily represent those of their affiliated organizations, or those of the publisher, the editors and the reviewers. Any product that may be evaluated in this article, or claim that may be made by its manufacturer, is not guaranteed or endorsed by the publisher.
